# Core‐binding factor acute myeloid leukemia with t(8;21): Risk factors and a novel scoring system (I‐CBFit)

**DOI:** 10.1002/cam4.1733

**Published:** 2018-08-16

**Authors:** Celalettin Ustun, Elizabeth Morgan, Erica E. M. Moodie, Sheeja Pullarkat, Cecilia Yeung, Sigurd Broesby‐Olsen, Robert Ohgami, Young Kim, Wolfgang Sperr, Hanne Vestergaard, Dong Chen, Philip M. Kluin, Michelle Dolan, Krzysztof Mrózek, David Czuchlewski, Hans‐Peter Horny, Tracy I. George, Thomas Kielsgaard Kristensen, Nam K. Ku, Cecilia Arana Yi, Michael Boe Møller, Guido Marcucci, Linda Baughn, Ana‐Iris Schiefer, J. R. Hilberink, Vinod Pullarkat, Ryan Shanley, Jessica Kohlschmidt, Janie Coulombe, Amandeep Salhotra, Lori Soma, Christina Cho, Michael A. Linden, Cem Akin, Jason Gotlib, Gregor Hoermann, Jason Hornick, Ryo Nakamura, Joachim Deeg, Clara D. Bloomfield, Daniel Weisdorf, Mark R. Litzow, Peter Valent, Gerwin Huls, Miguel‐Angel Perales, Gautam Borthakur

**Affiliations:** ^1^ Division of Hematology, Oncology and Transplantation Department of Medicine University of Minnesota Minneapolis Minnesota; ^2^ Department of Pathology Harvard Medical School Brigham and Women's Hospital Boston Massachusetts; ^3^ Department of Epidemiology, Biostatistics & Occupational Health McGill University Montreal Quebec Canada; ^4^ Department of Pathology University of California Los Angeles California; ^5^ Fred Hutchinson Cancer Research Center Seattle Washington; ^6^ University of Washington School of Medicine Seattle Washington; ^7^ Department of Dermatology and Allergy Centre Odense Research Center for Anaphylaxis Odense Denmark; ^8^ Mastocytosis Center Odense University Hospital Odense Denmark; ^9^ Department of Pathology Stanford University Stanford California; ^10^ Department of Pathology City of Hope National Medical Center Duarte California; ^11^ Division of Hematology & Hemostaseology Ludwig Boltzmann Cluster Oncology Department of Internal Medicine I Medical University of Vienna Vienna Austria; ^12^ Department of Hematology Odense University Hospital Odense Denmark; ^13^ Department of Pathology Mayo Clinic Rochester Minnesota; ^14^ Department of Pathology and Medical Biology University Medical Center Groningen University of Groningen Groningen The Netherlands; ^15^ Department of Laboratory Medicine and Pathology University of Minnesota Minneapolis Minnesota; ^16^ The Ohio State University Comprehensive Cancer Center Columbus Ohio; ^17^ Department of Pathology University of New Mexico Albuquerque New Mexico; ^18^ Institute of Pathology Ludwig‐Maximilians‐University Munich Germany; ^19^ Department of Pathology University of Utah Salt Lake City Utah; ^20^ Department of Pathology Odense University Hospital Odense Denmark; ^21^ Division of Hematology and HCT City of Hope Duarte California; ^22^ Department of Pathology Medical University of Vienna Vienna Austria; ^23^ Biostatistics and Bioinformatics University of Minnesota Minneapolis Minnesota; ^24^ Alliance Statistics and Data Center The Ohio State University Comprehensive Cancer Center Columbus Ohio; ^25^ Department of Medicine Adult Bone Marrow Transplant Service Memorial Sloan Kettering Cancer Center New York NY; ^26^ Department of Medicine Weill Cornell Medical College New York New York; ^27^ Division of Allergy and Clinical Immunology University of Michigan Ann Arbor Michigan; ^28^ Stanford Cancer Institute School of Medicine Stanford University Stanford California; ^29^ Department of Laboratory Medicine Medical University of Vienna Vienna Austria; ^30^ Department of Internal Medicine and Division of Hematology Mayo Clinic Rochester Minnesota; ^31^ Department of Hematology University Medical Center Groningen University of Groningen Groningen The Netherlands; ^32^ Department of Leukemia University of Texas M.D. Anderson Cancer Center Houston Texas

**Keywords:** acute myeloid leukemia, core‐binding factor, disease‐free survival, *KIT* mutation, predictive value, relapse, scoring system

## Abstract

**Background:**

Although the prognosis of core‐binding factor (CBF) acute myeloid leukemia (AML) is better than other subtypes of AML, 30% of patients still relapse and may require allogeneic hematopoietic cell transplantation (alloHCT). However, there is no validated widely accepted scoring system to predict patient subsets with higher risk of relapse.

**Methods:**

Eleven centers in the US and Europe evaluated 247 patients with t(8;21)(q22;q22).

**Results:**

Complete remission (CR) rate was high (92.7%), yet relapse occurred in 27.1% of patients. A total of 24.7% of patients received alloHCT. The median disease‐free (DFS) and overall (OS) survival were 20.8 and 31.2 months, respectively. Age, *KIT* D816V mutated (11.3%) or nontested (36.4%) compared with *KIT* D816V wild type (52.5%), high white blood cell counts (WBC), and pseudodiploidy compared with hyper‐ or hypodiploidy were included in a scoring system (named I‐CBFit). DFS rate at 2 years was 76% for patients with a low‐risk I‐CBFit score compared with 36% for those with a high‐risk I‐CBFit score (*P *< 0.0001). Low‐ vs high‐risk OS at 2 years was 89% vs 51% (*P *< 0.0001).

**Conclusions:**

I‐CBFit composed of readily available risk factors can be useful to tailor the therapy of patients, especially for whom alloHCT is not need in CR1 (ie, patients with a low‐risk I‐CBFit score).

## 1 INTRODUCTION

Acute myeloid leukemia (AML) with rearrangements involving genes encoding subunits of core‐binding factor (CBF), a group of DNA‐binding transcription factor complexes composed of α and β subunits, shares similar pathogenesis and clinical features and is considered as a distinct subset in AML.^1^, ^2^, ^3^, ^4^ Translocation(8;21)(q22;q22) and inv(16)(p13q22), the most frequent cytogenetic abnormalities occurring in CBF‐AML, lead to the creation of the fusion genes *RUNX1/RUNXT1* and *CBFB/MYH11* that disrupt, respectively, the α and β subunits of CBF, dysregulate hematopoiesis, and thus contribute to leukemogenesis.^5^

Although the prognosis of CBF‐AML is better than other subtypes of AML, approximately 30%‐40% of the patients still relapse and may require allogeneic hematopoietic cell transplantation (HCT).^6^, ^7^, ^8^ A scoring system to predict who has a higher risk of relapse at the time of diagnosis may be clinically valuable to guide decision‐making. There have been only a few studies attempting to develop a scoring system for poor outcomes of CBF‐AML (eg, relapse and disease‐free survival [DFS]).^6^, ^8^ The relative rarity of CBF‐AML (approximately 15%‐20% of AML cases) in adults^9^ and its relatively good prognosis may have limited these efforts. A useful prognostic system requires a large sample size and long follow‐up time including all treatment data. This is challenging, even for large registries or cooperative groups. For example, the Center for International Blood and Marrow Transplant Research (CIBMTR) only has data of patients with CBF‐AML receiving a HCT, while US cooperative groups may have too few patients with a long follow‐up to examine outcomes after HCT. Moreover, recent studies clearly indicate that AMLs with t(8;21) (q22;q22) and AMLs with inv(16) (p13q22) are two different diseases regarding patient and disease characteristics.^2^, ^6^, ^8^, ^10^, ^11^, ^12^, ^13^, ^14^ Each cytogenetic subgroup therefore should be evaluated separately to develop a specific prognostic scoring system.

In this multicenter study, we created an extensive database including US and European centers for CBF‐AML patients with t(8;21) (q22;q22), and developed and validated a significant risk scoring system with high predictive probabilities.

## 2 METHODS

Eleven centers in the US and Europe collaborated to collect data on 550 CBF‐AML patients. Two‐hundred and forty‐seven of these patients had t(8;21)(q22;q22) and are the subject of this report. Inclusion criteria were as follows: (a) AML patients with t(8;21)(q22;q22) or *RUNX1‐RUNX1T1* confirmed by the reporting institutions; (b) cases diagnosed between July 1996 and January 2017. Data were uniformly collected by completing a predesigned data spreadsheet. The data form included the following: patient characteristics (age, sex, race); disease characteristics (date of diagnosis, white blood cell count [WBC] at diagnosis [×10^9^/L], cytogenetics, *KIT* D816V mutational status, primary or secondary AML); therapy characteristics (induction regimens and their number, consolidation regimens, and number of cycles); HCT (autologous or allogeneic, donor type, remission status at HCT); and events (relapse, death, or alive at last contact). Patients’ data were anonymously transferred to University of Minnesota where the main database was created and managed. This study was approved by the Institutional Review Board Human Subjects Committee at the University of Minnesota.

### 2.1 Definitions

Secondary AML was assigned if a patient had a history of chemotherapy/radiation therapy for a malignancy and/or had a history of preleukemic disease (eg, myelodysplastic syndrome [MDS], myeloproliferative neoplasm [MPN]). In cytogenetic evaluation, a total number of 46 chromosomes were defined as pseudodiploidy in one clone or each clone (given this patients had translocation, it was not named diploidy), and if chromosome number was higher or lower than 46 chromosomes in any clone, it was defined, respectively, as hyperdiploidy and hypodiploidy.

### 2.2 Statistical analysis

The sample of 247 patients was described using the median and range for continuous variables, and frequency and percentage for categorical variables.

The binary outcome was defined as death or relapse within 2 years of diagnosis. A total of 89 patients experienced death or relapse within 2 years, while 158 patients survived without relapse or were censored at the last contact alive (or in remission).

A set of potential predictors for our outcome of relapse‐free survival was selected to build the risk score model, which were used to predict the probability of death or relapse in 2 years. The predictors included age, sex, race (Caucasian), WBC at diagnosis, ‐X, ‐Y, chromosome 5 or 7 abnormalities, chromosome 4 abnormalities, chromosome 9 abnormalities, trisomy 8, number of chromosomes, *KIT* D816V mutation, and primary AML. The missing values for the variable *KIT* D816V mutation were combined into the category nontested instead of imputing the variable, so as to allow risk prediction when this variable is missing. The remaining covariates that had missing values in the dataset were variables considered unlikely to be missing in clinical practice, and thus, multiple imputation was used so as to construct a clinically meaningful risk score that made full use of available patient information.

Full details of the statistical analysis are provided in the Appendix S1. In brief, forward stepwise logistic regression was used, with the binary outcome of two‐year relapse or death and the predictors discussed above. The optimal threshold for binary predictions was chosen to maximize equally the sensitivity and specificity. A validation study was used to assess the performance of the risk score model using fivefold cross‐validation to estimate specificity, sensitivity, accuracy, positive predictive value (PPV), and negative predictive value (NPV).

We performed three sensitivity analyses. In the first, patients were censored upon allogeneic HCT (alloHCT) at CR1, as this is not a standard therapy. In the second, we considered only survival (rather than disease‐free survival). In the final sensitivity analysis, we imputed all missing values (including *KIT* D816V mutation) to create a risk score that would require all relevant covariates to be observed rather than allowing for the possibility that some are unavailable to the clinician.

## 3 RESULTS

The characteristics of the test and validation groups combined are provided in Table 1. Patients were mostly male, were Caucasian, and had a median age of 47 years, and 17.4% had secondary AML. Additional cytogenetic abnormalities were frequently observed (58.7%), and 44.5% of patients had a hypodiploid or hyperdiploid clone. *KIT* D816V mutation was present in 11.3% of patients (17.8% of the patients tested), and any *KIT* mutations were detected in 16.6% of patients (25.7% of the patients tested). There was no association between KIT mutation (positive, negative, nontested) and WBC (Figure S1).

**Table 1:** Characteristics of patients

Variable	Total
Number	247
Age, median (range) y	47.0 (2.0‐81.0)
Missing, n (%)	1 (0.4%)
Sex, n (%)	
Female	101 (40.9%)
Male	132 (53.4%)
Missing, n (%)	14 (5.6%)
Race, n (%)	
Caucasian	176 (71.3%)
Other	48 (19.4%)
Missing, n (%)	23 (9.3%)
Year of diagnosis, median (range)	2009 (1995‐2017)
Missing, n (%)	2 (0.8%)
WBC at diagnosis, median (range) ×10^9^/L	11.7 (1.3‐139.9)
Missing, n (%)	19 (7.7%)
AML, n (%)	
Primary	194 (78.5%)
Secondary	43 (17.4%)
Missing, n (%)	10 (4.0%)
Cytogenetics	
‐X, n (%)	
No	206 (83.4%)
Yes	33 (13.4%)
Missing, n (%)	8 (3.2%)
‐Y, n (%)	
No	192 (77.7%)
Yes	48 (19.4%)
Missing, n (%)	7 (2.8%)
Chromosome 9 abnormalities, n (%)	
No	210 (85.0%)
Yes	29 (11.7%)
Missing, n (%)	8 (3.2%)
Chromosome 4 abnormalities, n (%)	
No	232 (94.0%)
Yes	7 (2.8%)
Missing, n (%)	8 (3.2%)
Chromosome 5 or 7 abnormalities, n (%)	
No	210 (85.0%)
Yes	28 (11.3%)
Missing, n (%)	9 (3.6%)
+8, n (%)	
No	211 (85.4%)
Yes	28 (11.3%)
Missing, n (%)	8 (3.2%)
Number of Chromosomes, n (%)	
46	129 (52.2%)
<46	87 (35.2%)
>46	23 (9.3%)
Missing, n (%)	8 (3.2%)
Additional cytogenetic abnormality, n (%)	
Yes	145 (58.7%)
No	95 (38.5%)
Missing, n (%)	7 (2.8%)
KIT mutation, n (%)	
Negative	118 (47.8%)
Positive	41 (16.6%)
Nontested/Missing, n (%)	88 (35.6%)
KIT D816V mutation, n (%)	
Negative	129 (52.5%)
Positive	28 (11.3%)
Nontested/Missing	90 (36.4%)
CR status, n (%)	
Yes	229 (92.7%)
Relapse, n (%)	
Yes	67 (27.1%)
Missing, n (%)	1 (0.4%)
Does not apply (%)	18 (7.3%)
AlloHCT, n (%)	
Yes	61 (24.7%)
Disease status at alloHCT n (%)	
No CR	10 (4.0%)
CR1	31 (12.5%)
CR2	18 (7.3%)
>CR2	1 (0.4%)
Missing	1 (0.4%)
Does not apply	186 (75.3%)
DFS, median (range) mo	20.8 (0‐225.8)
Missing, n (%)	1 (0.4%)
OS, median (range) months	31.2 (1‐245.8)
Missing, n (%)	0 (0.0%)

AlloHCT, allogeneic hematopoietic cell transplantation; CR, complete remission; DFS, disease‐free survival; OS, overall survival; WBC, white blood cell count.

Complete remission (CR) was achieved in the vast majority of patients (92.7) (Table 1). Relapse occurred in 67 patients (27.1%) at a median of 10.6 months (range 1‐65.5 months). AlloHCT was performed in 61 patients (24.7%): 31 with CR1 (12.5%), 19 with ≥CR2 (7.6%), and 10 (4.0%) with active leukemia (all relapsed after CR). AlloHCT in CR1 was performed at a median of 6 months (range 2 to 13.1 months) from diagnosis and 4 months (1.1‐12 months) from the date of CR1. The median follow‐up was 64 months (0.5 to 1378 months).

The risk factors and risk ratios from a logistic regression model are presented in Table 2. Older age, higher WBC at diagnosis, *KIT* D816V mutation, and a pseudodiploid karyotype were associated with higher risks of death or disease relapse. Race, sex, and primary vs. secondary AML had no impact.

**Table 2:** Risk ratios of risk factors for death or relapse

Risk factor	Risk ratio	*P*‐value
Age	1.031	0.0017
*KIT* D816V mutation positive (Ref = negative)	4.331	0.0018
*KIT* D816V mutation nontested/missing (Ref = negative)	2.567	0.0036
WBC at diagnosis	1.018	0.0361
Number of chromosomes (Ref = nonpseudodiploidy)	2.552	0.0035

WBC indicates white blood cell count.

The risk of death or relapse within 2 years associated with the covariates retained in the predictive risk score is shown in Table 2. The concordance statistic (a measure of the model fit, also called the area under curve (AUC), or area under the receiver operating characteristics (ROC) curve for the predictions) is 0.756 (Figure S2). The optimal risk score is found by computing the following linear score:

$$\begin{array}{cc} I\text{‐}\text{CBFIT Score}= & -3.05\\ & +0.03\text{Age years}\\ & +0.02\;\text{WBC at diagnosis}\;(\times 10^{9}/L)\\ & +1.47(KIT\;\text{D816V mutation positive})\\ & +0.94(KIT\;\text{D816V mutation nontested}/\text{missing})\\ & +0.94(\text{pseudodiploidy}) \end{array}$$

**Figure 1:**
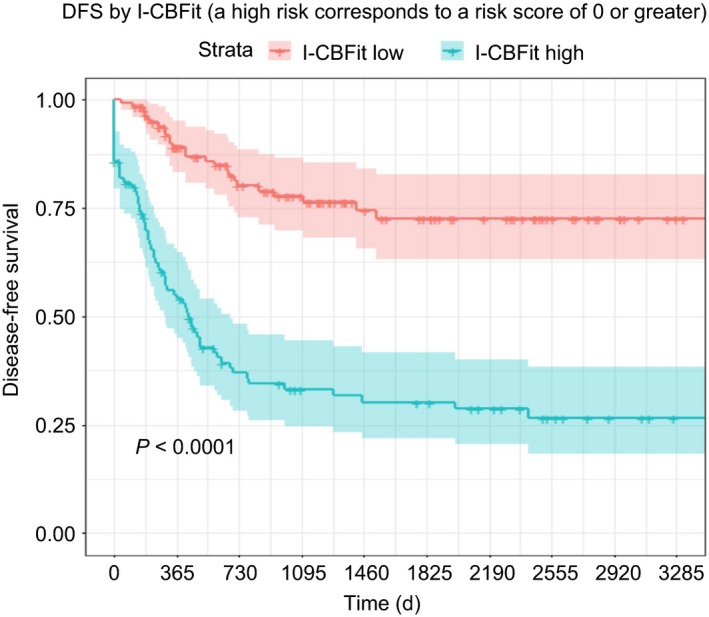
Patients with a low I‐CBFit score (red curve with 95% CI) had significantly higher DFS compared with those who had a higher score (green curve with 95% CI)

**Figure 2:**
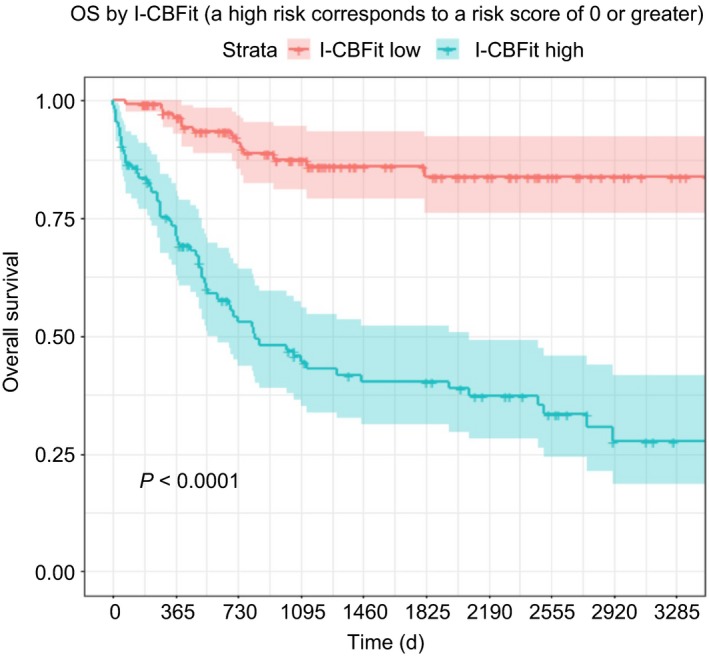
Patients with a low I‐CBFit score (red curve with 95% CI) had significantly higher OS compared with those who had a higher score (green curve with 95% CI)

The full set of results of the validation study along with the sensitivity analysis results (the highest of the conditional probabilities was negative predictive value [NPV], 80%) are presented in Table S1. When I‐CBFit > 0, a patient is classed as being at high risk of death or relapse within 2 years. DFS rate at 2 years was 76% for patients with a low‐risk I‐CBFit score compared with 36% for those with a high‐risk I‐CBFit score (*P *<* *0.0001). Low‐ vs high‐risk OS at 2 years was 89% vs 51%, *P* < 0.0001 (Figures 1 and 2).

**Figure 3:**
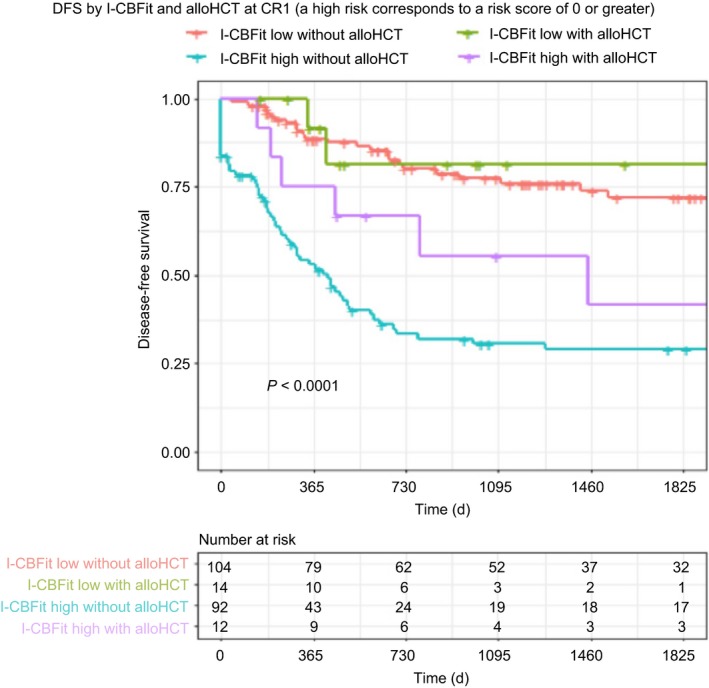
DFS is stratified by alloHCT and I‐CBFit score. AlloHCT did not have an impact on DFS in patients with a low I‐CBFit score (red and green curves); however, patients with high I‐CBFit‐risk had improved DFS after alloHCT compared with those who did not undergo alloHCT (purple and green curves)

**Figure 4:**
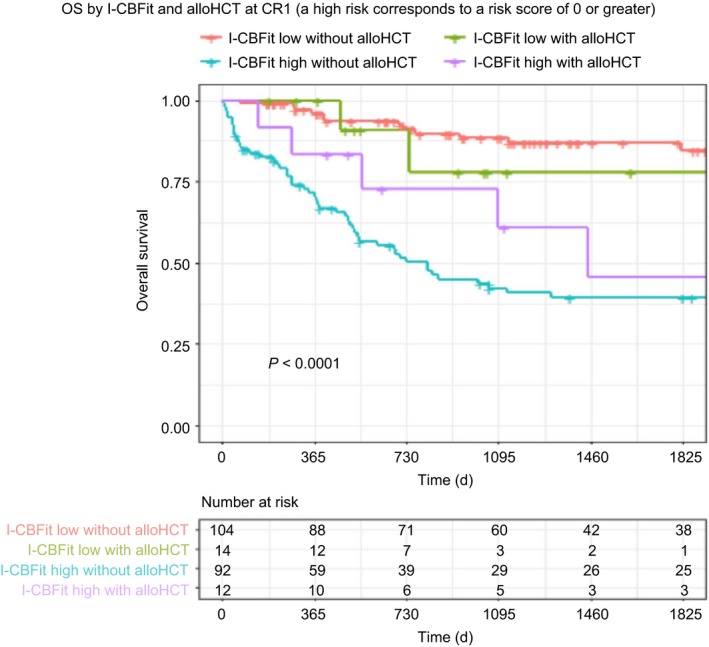
OS is stratified by alloHCT and I‐CBFit score. AlloHCT did not have an impact on OS in patients with a low I‐CBFit score (red and green curves); however, patients with high I‐CBFit risk had improved OS after alloHCT compared with those who did not undergo alloHCT (purple and green curves)

DFS at 2 years was 80% for patients with I‐CBFit low risk not undergoing alloHCT in CR1, was 82% for patients with I‐CBFit low risk undergoing alloHCT in CR1, was 33% for patients with I‐CBFit high risk not undergoing alloHCT in CR1, and was 67% for patients with I‐CBFit high risk undergoing alloHCT in CR1, *P* = <0.0001 (Figure 3). OS at 2 years was 91% for patients with I‐CBFit low risk regardless of alloHCT in CR1, was 52% for patients with I‐CBFit high risk not undergoing alloHCT in CR1, and was 73% for patients with I‐CBFit high risk undergoing alloHCT in CR1, *P* < 0.0001 (Figure 4).

## 4 DISCUSSION

In this large study with a long follow‐up, we were able to create and validate the risk scoring system we are calling the “International CBF group index for t(8;21)” (I*‐*CBFit) in t(8;21) AML. We show that older age, higher WBC at diagnosis, and *KIT* D816V mutation were risk factors associated with treatment failure (relapse or death). In addition, we found that pseudodiploidy was also a risk factor in t(8;21), a novel finding. I‐CBFit had a high NPV (80%) and a modest specificity and accuracy for DFS, and the NPV was even higher for the prediction of OS.

Current treatment guidelines for CBF‐AML with t(8;21) do not recognize heterogeneity in these patients, and thus, all t(8;21) AML patients generally receive the same induction and consolidation treatments. This might be appropriate for patients with a low‐risk score who are predicted to have nearly an 80% chance of extended DFS. On the other hand, high‐risk score patients may benefit from more intensive approaches in CR1. Current guidelines do not identify patients needing alloHCT in CR1. This new model may clarify this uncertainty, especially identifying patients who do not require intensive consolidations (eg, alloHCT) in CR1 given its high NPV. Although patients receiving alloHCT in CR1 was limited, when we analyzed the impact of alloHCT it seemed that patients with an I‐CBFit low‐risk score had similar DFS and OS regardless of alloHCT.

*KIT* mutations have been reported in 15%‐46% of adults patients with t(8;21) CBF‐AML.^13^, ^15^, ^16^, ^17^, ^18^ *KIT* D816V mutations were reported in 4%‐28% and strongly associated with poorer DFS (6%‐48%).^13^, ^16^, ^19^, ^20^ In pediatric populations, *KIT* mutations clustered in exon 17 and exon 8 were identified in 20‐30% of the CBF‐AML patients,^21^, ^22^, ^23^ yet its effect on prognosis is not agreed upon.^22^, ^24^ A meta‐analysis indicated *KIT* mutation increased relapse risk (RR at 2 years 1.76 [95% CI: 1.45‐2.12]) and decreased OS 1.35 (95% CI: 1.09‐1.66).^25^

Chromosomal abnormalities secondary to t(8;21), mostly involving loss of a sex chromosome, ‐Y in men and ‐X in women, trisomy 8, and deletion of the long arm of chromosome 9 [del(9p)] are frequently reported.^6^, ^8^, ^14^, ^18^ In our patients, additional cytogenetic abnormalities were common, as in other reports.^14^, ^18^, ^26^ Sex chromosome loss was reported as favorable for two‐year event‐free survival (66.9% vs 43.0%, *P *=* *0.031).^18^ In contrast, DFS was shorter for male patients with loss of the Y chromosome. In another study,^8^ loss of a sex chromosome was associated with increased CR rates in CBF‐AML.^14^ We found no particular chromosomal abnormality to be associated with poor outcome. However, consistent with findings of Krauth et al^18^ loss of a sex chromosome had a modestly favorable, but not significant effect on DFS. We also found that the chromosome number was important, with patients with pseudodiploid karyotypes having worse outcome compared with those with hypodiploidy or hyperdiploidy.

Higher WBCs were found to be associated with poorer outcomes.^8^ Schlenk et al^8^ described a scoring system using two factors, high WBC, and low platelet counts, to be prognostic. Low platelet count was also a poor prognostic factor in a CALGB/Alliance study.^6^ In our study, we did not find a correlation between *KIT* mutation and WBC.

An earlier CALGB/Alliance study showed that age was associated with shorter overall survival (OS).^6^ In a more recent CALGB/Alliance study,^27^ 3‐year OS rate was 61% for adults younger than 60 years vs only 47% for those at least 60 years old. Appelbaum et al^14^ showed that age is associated with a shorter OS.

We were able to collect data over a two‐decade period and believe this long time period does not adversely impact the validity of the study as (a) the type and number of induction or consolidation therapies did not have an impact on outcomes and (b) the most widely used treatments (7 + 3 in induction phase and high‐dose cytarabine in consolidation phase) have not changed over this time. Although this is a retrospective study, we find the data robust and substantial given the lengthy time period of patient follow‐up. In fact, long‐term follow‐up allowed complete evaluation in this relatively good prognostic disease.

Another limitation is that molecular abnormalities, including mutations in the *KIT* and *FLT3* genes, were not uniformly tested. As a result, information on *KIT* mutational status is missing in approximately one‐third of the patients. However, *KIT* mutation was associated with significantly decreased survival compared with *KIT* wild type, whereas outcomes of patients with the *KIT* mutational status untested fell between outcomes of patients with *KIT* mutations and those with wild‐type *KIT;* this might be expected given that some but not all untested patients would have mutated *KIT*. This strongly supports the adverse effect of a *KIT* mutation.

This new scoring system, I‐CBFit, uses known and novel risk factors to provide a binary prediction of the risk of death or relapse within 2 years. Importantly, all factors and thus the scoring system can easily be determined at diagnosis. Although its validation by other studies is needed, I‐CBFit can contribute to current treatment of patients with t(8;21) and tailor consolidation treatments for individual patients in the spirit of precision medicine to identify those who do not need intensified management including alloHCT during CR1.

## CONFLICT OF INTEREST

The authors have no conflict of interest relevant to the study to disclose.
