# Supplementary figures and images for: Core‐binding factor acute myeloid leukemia with t(8;21): Risk factors and a novel scoring system (I‐CBFit)
Source: Cancer Med. 2018 Aug 16;7(9):4447–55. doi: 10.1002/cam4.1733 (PMC6144246; doi:10.1002/cam4.1733)

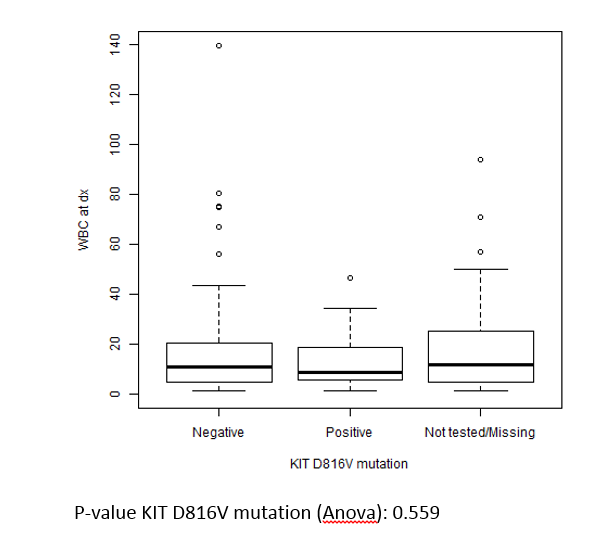

Supplement: Supplementary file 1 [file CAM4-7-4447-s001.PNG]

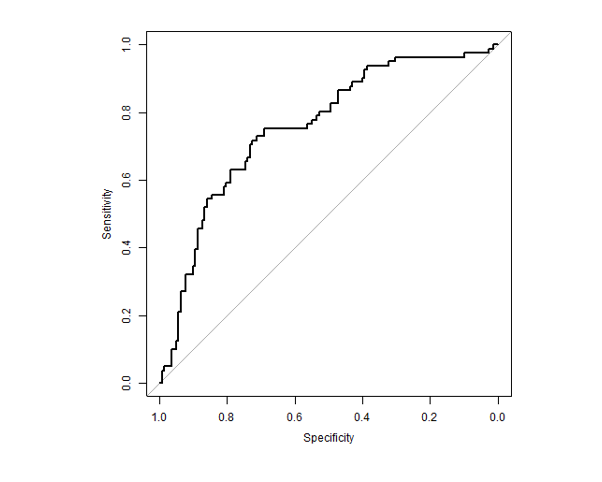

Supplement: Supplementary file 2 [file CAM4-7-4447-s002.PNG]
