# Supplementary material for: Core‐binding factor acute myeloid leukemia with t(8;21): Risk factors and a novel scoring system (I‐CBFit)
Source: Cancer Med. 2018 Aug 16;7(9):4447–55. doi: 10.1002/cam4.1733 (PMC6144246; doi:10.1002/cam4.1733)
Supplement: Supplementary file 3 [file CAM4-7-4447-s003.docx]

***Online Web Supplement for***

**Core-Binding Factor Acute Myeloid Leukemia with t(8;21): Risk Factors and A Novel Scoring System (I-CBFit)**

**Further details of the statistical analysis:**

Missing data were handled via multiple imputation with chained equations (MICE). We built 25 replicates of a complete dataset so as to predict a risk score that made full use of available data with clinically meaningful and available patient information. These replicates ensure the variability due to the imputation process is adequately incorporated into the standard errors and other reported inferences.^1^ Forward stepwise logistic regression was fitted on each of the 25 replicated datasets, with the binary outcome two-year treatment-failure and the predictors discussed above. The final set of predictors for the risk score included the covariates retained at least once, and a pooled logistic regression was fitted across all 25 replicated datasets, using that final set of predictors. A receiver-operator characteristics curve^2^ was plotted and the optimal threshold for binary predictions was chosen so as to maximize equally the sensitivity and specificity (**Supplemental Figure 1**).

A validation study was used to assess the performance of the risk score model using five-fold cross validation was performed on the full sample, splitting the observations in five equal parts and using each time one fifth as the testing dataset and the remaining four fifths parts as the training dataset. Specificity, sensitivity, accuracy, positive predictive value (PPV) and negative predictive value (NPV) were thus computed in each of the 5 testing sets and averaged to produce estimates of the model accuracy (**Supplemental Table 1**).

**Supplemental Table 1:** Validation of the model with 5-fold cross validation (mean statistics) for the main analysis, and three sensitivity analyses

|  | **Main analysis on DFS** | **Sensitivity analysis: Censoring at Allo HCT at CR1** | **Sensitivity analysis: Death as outcome (OS)** | **Sensitivity analysis: imputed missing data** |
| --- | --- | --- | --- | --- |
| **Sensitivity** | 68% | 64% | 55% | 67% |
| **Specificity** | 71% | 66% | 74% | 68% |
| **Accuracy** | 71% | 67% | 69% | 67% |
| **Positive predictive value** | 58% | 50% | 40% | 55% |
| **Negative predictive value** | 80% | 81% | 83% | 78% |

AlloHCT indicates allogeneic hematopoietic cell transplantation; DFS, disease-free survival; OS, overall survival

**References:**

1. Van Burren S. Multiple imputation of discrete and continuous data by fully conditional specification. Stat Methods Med Res 2007;16(3):219-42.
2. Hanley JA, McNeil BJ. The meaning and use of the area under a receiver operating characteristics (ROC) curve. Radiology 1982;143(1):29-36.
